# How Autonomy Support Sustains Emotional Engagement in College Physical Education: A Longitudinal Study

**DOI:** 10.3390/bs15060822

**Published:** 2025-06-15

**Authors:** Qifei Xia, Shu Xuan, Tingxiao Zhang, Bobo Zong

**Affiliations:** 1 School of Physical Education, Ankang College, Ankang 725000, China; xiaqifei@aku.edu.cn; 2 School of Physical Education, Hunan University, Changsha 410082, China; xuanshu@hnu.edu.cn; 3 School of Physical Education, Liaoning Normal University, Dalian 116029, China; zhangtx0723@lnnu.edu.cn; 4 School of Physical Education China University of Geosciences, Wuhan 430074, China; 5 School of Physical Education, Beijing Normal University, Beijing 100875, China

**Keywords:** autonomy support, emotional engagement, physical education, longitudinal study, self-determination theory

## Abstract

Traditional educational models and assessment tools have neglected the motivation and sustainability of students’ emotional engagement in physical education classes. The impact of self-support on emotional engagement in physical education classes and its underlying mechanisms remain under-explored. The present study was a 6-month longitudinal study of 718 college students at two universities in a remote area of southern Shaanxi Province, China. The study aimed to examine the effects of autonomy support on affective engagement in physical education classes and to analyze the longitudinal mediating roles of self-acceptance and academic self-efficacy. The study found that physical education teacher support and parental autonomy support significantly promoted college students’ emotional engagement in physical education classes. Chained longitudinal mediation analyses indicated that self-acceptance and academic self-efficacy played chained mediating roles in promoting college students’ affective engagement in physical education classes in different supportive environments. This study transcends a static, cross-sectional research perspective, validates and extends self-determination theory, and promotes affective engagement in physical education learning through different autonomy supports that enhance the development of self-acceptance and academic self-efficacy in college students, providing a reference for enhancing physical education teaching and learning and improving the quality of teaching and learning.

## 1. Introduction

The concept of a high-quality “physical education curriculum” has long been a focal point in educational practice. This issue is equally pertinent across disciplines, including education, psychology, and public health (Dudley & Burden, 2020). Physical education differs from other subjects by its emphasis on students’ acquisition of physical knowledge and skills, improvement of lifestyle, and development of social cognition and emotional experiences. As educational reforms in physical education continue worldwide, it is imperative to explore how to enhance teaching quality and effectiveness. Student engagement refers to active participation in learning activities, deep thinking, and energetic responses to challenges and setbacks (Reschly & Christenson, 2022). The term “engagement” was first applied to the learning process by Pascarella and Terenzini (Pascarella & Terenzini, 1991), and Bevans later contextualized it within physical education, highlighting individual efforts to master knowledge and skills, manifesting as preferences for physical activities and active participation in learning processes (Bevans et al., 2010). Engagement encompasses three dimensions: behavioral, emotional, and cognitive, which interact and collectively influence learning outcomes (Garn et al., 2017). Among these, emotional engagement plays a crucial role in physical education, encompassing learners’ affective experiences, such as interest, enthusiasm, and positive emotions (Fredricks et al., 2004; Skinner & Belmont, 1993). Emotional engagement, as an endogenous factor supporting learning, is one of the most important factors in promoting students’ learning behavior and driving their cognitive engagement (Schutz & Lanehart, 2002). Especially in the process of practical learning in the physical education classroom, higher affective engagement is usually characterized by interest and pleasure in the classroom, recognition of the value of health promotion, and a sense of belonging to a team, which means that affective engagement is crucial for enhancing students’ performance in physical education (Wiedenman et al., 2024).

Research suggests that highly emotionally engaged learners display positive emotions, such as happiness, while those with lower emotional engagement tend to avoid challenges or face them negatively, lacking the motivation to exert effort (Connell, 1991). Increasing emotional engagement in physical education is essential for enhancing students’ proactive learning attitudes and cultivating healthy exercise habits, ultimately improving teaching quality. However, insufficient emotional engagement in university physical education may be particularly prominent (Baños et al., 2023). It has been demonstrated that teacher support can influence student classroom engagement by increasing students’ positive emotions (T. Zhang et al., 2024). When teachers are aware of the role of emotions in their teaching practice, empathy for their students is usually enhanced as well (Zach & Rosenblum, 2021). A study of university students in the southern United States revealed that differences in the content, format, and difficulty of physical education could influence students’ emotional responses (e.g., anxiety, stress, and anger) and even affect their GPA (Dobersek & Arellano, 2017). Another study indicated that students who perceive the knowledge and skills learned in physical education as valuable, rewarding, and predictive of academic success tend to experience higher levels of enjoyment, excitement, and enthusiasm (Garn et al., 2017).

In the Chinese educational context, university physical education often suffers from a lack of health education, minimal physical activity load, limited variety of activities, and a strong focus on performance in competitive settings. This results in a lack of interest, disengagement, boredom, fatigue, and poor learning outcomes among students (Wu et al., 2022). In comparison to Western countries, the “hands-off” teaching approach in Chinese physical education has become a major barrier to fostering students’ interest in physical activity and promoting physical health, which may adversely affect their academic performance and social adaptation. Therefore, it is essential to identify the underlying factors influencing emotional engagement in physical education and to develop effective strategies for intervention. In particular, within China’s high-pressure educational environment, addressing the factors that impact emotional engagement in physical education is crucial to enhancing students’ academic quality and future career prospects.

## 2. Literature Review and Research Hypotheses

### 2.1. Autonomy Support and Emotional Engagement in Physical Education

The concept of “autonomy” has deep historical roots and is an integral aspect of personality development in Chinese culture (Xia et al., 2013). It is widely applied across various domains, including individual, group, political, and economic contexts. While similar to the Western concept of “independence,” autonomy, like independence, is a key personality trait and a complex construct. Autonomy refers to the process of individuals freeing themselves from prior dependencies (Huang & Li, 2001), typically supported by family or educational environments that provide choices, respect individual opinions, and encourage autonomous decision-making. According to Deci and Ryan’s Self-Determination Theory (SDT) (E. L. Deci & Ryan, 1985), intrinsic motivation and the internalization of external behaviors are influenced by environmental factors. When an environment fosters autonomy, individuals are more likely to engage actively in activities. The concept of autonomy support originates from SDT, with teacher autonomy support being a specific application within physical education contexts. Autonomy support from teachers is widely recognized as a critical external factor influencing deep learning among university students (Kaplan, 2018). Stefanou et al. (Stefanou et al., 2004) developed a theoretical framework identifying three distinct dimensions of teacher autonomy support in physical education: the cognitive dimension (encouraging students to express and debate their viewpoints), the process dimension (empowering students to take ownership of learning processes), and the organizational dimension (involving students in decisions regarding the classroom environment). Students’ perceptions of autonomy support from physical education teachers is a positive predictor of intrinsic motivation to learn in the physical education classroom (Arik & Erturan, 2023). For physical education learners, teacher autonomy support fosters emotional recognition, as students feel supported and encouraged to make autonomous decisions and exercise freedom in their learning (Ryan, 2001). There is research evidence that physical education teachers use highly autonomous and supportive teaching styles (e.g., through a combination of free choice, social interaction, and informational feedback) to enhance students’ positive affective perceptions and increase the amount of time students spend engaging in physical activity, providing evidence-based strategies for autonomy support (Leisterer & Paschold, 2022). In contrast to controlling teachers, those who provide autonomy support use non-controlling language, empathize with students’ challenges in learning physical skills, acknowledge their perspectives, and offer meaningful choices and new tasks (White et al., 2021). In this regard, this autonomy support can lead to a higher level of classroom well-being, stimulate their motivation to learn independently (Chrov & Ran, 2001), and influence academic achievement through classroom enjoyment and classroom satisfaction (Baños et al., 2020).

The family plays a central role in an individual’s social support system and significantly influences students’ emotional engagement in learning. Parental autonomy support refers to parents respecting their children’s autonomy, accepting their emotions and thoughts, and minimizing control, while encouraging independent decision-making (Weinstein et al., 2012). This support enables children to explore and practice their values and interests, particularly in physical education (Wang et al., 2007). Studies have shown that children with more parental support are more enthusiastic and engaged in sports like soccer (De Muynck et al., 2021). According to SDT (Vansteenkiste & Ryan, 2013), supportive environments satisfy three basic psychological needs, autonomy, relatedness, and competence, while controlling environments have the opposite effect. Parental autonomy support promotes motivation and helps children maintain positive psychological states, contributing to greater engagement in physical education (Vasquez et al., 2016). This view is supported by Gillet et al. (Gillet et al., 2012), who found that a trusting and encouraging family atmosphere fosters emotional support, enhancing students’ engagement in physical education.

Although much research focuses on assessing and adjusting academic engagement, fewer studies have addressed the dynamic nature of emotional engagement in physical education. Moreover, there is limited exploration of the relationship between autonomy support and emotional engagement in physical education. Therefore, based on the existing literature and to fill this gap, we propose the following hypotheses:

**H1a.**  *Teacher autonomy support significantly predicts emotional engagement in physical education among university students*.

**H1b.**  *Parental autonomy support significantly predicts emotional engagement in physical education among university students*.

### 2.2. Mediating Role of Self-Acceptance

Self-acceptance refers to an individual’s conscious decision to embrace both positive and negative aspects of themselves or others (Williams & Lynn, 2010), which includes acknowledging both favorable and unfavorable self-evaluations (Meireles et al., 2021). It typically results from a balanced self-assessment (Carson & Langer, 2006). Early rational-emotive therapy emphasized unconditional self-acceptance, advocating for an objective view of one’s imperfections. Humanistic psychologists further assert that self-acceptance is a product of self-discovery and personal growth. Studies have shown that students who perceive autonomy support from physical education teachers not only embrace the teacher’s teaching methods but also engage in self-reflection, thereby enhancing their learning outcomes (Shen et al., 2009). Teachers’ explanations, demonstrations, feedback, and supportive expressions of warmth and encouragement enable students to recognize their strengths and achieve a more objective self-evaluation (Perlman & Webster, 2011), which may enhance emotional engagement in physical education.

Parental autonomy support also plays a crucial role in fostering self-acceptance. Research indicates that parental warmth and emotional support promote positive self-acceptance in children (Ma & Wang, 2015). Positive interactions and reliable, warm relationships with caregivers facilitate children’s self-evaluation and self-worth (Feiring & Taska, 1996). When parents provide valuable guidance, such as highlighting the relevance of a sport for career development, students are likely to reflect on their personal growth and potential, leading to greater engagement and positive academic outcomes (Pihu & Hein, 2007; Mammadov & Schroeder, 2023). From a developmental perspective, self-acceptance is a crucial trait that promotes psychological and social growth, playing an important role in the self-development of university students (X. Zhang et al., 2022). Individuals with high self-acceptance are more likely to appreciate their strengths, acknowledge their shortcomings, and understand the gap between ideal and realistic expectations (F. Liu et al., 2024). Consequently, these individuals are more inclined to engage in self-reflection and hands-on practice, which enhances their emotional engagement in physical education. Therefore, we hypothesize the following:

**H2a.**  *Self-acceptance mediates the relationship between teacher autonomy support and emotional engagement in physical education*.

**H2b.**  *Self-acceptance mediates the relationship between parental autonomy support and emotional engagement in physical education*.

### 2.3. Mediating Role of Academic Self-Efficacy

Academic self-efficacy refers to an individual’s belief in their ability to complete specific learning tasks (Bandura, 1986), which serves as a key determinant of learning engagement (Fredricks et al., 2004). Previous research has shown that academic self-efficacy mediates the relationship between perceived autonomy support and academic success among university students (Gutiérrez & Tomás, 2019). In physical education, when teachers provide students with autonomy in decision-making, students are more likely to choose activities that interest them, leading to greater persistence in achieving their learning goals and sustained effort (Guo et al., 2024). For instance, a longitudinal study demonstrated that students who perceive more autonomy support from teachers develop more autonomous forms of motivation and higher academic self-efficacy, making them less likely to experience dropout intentions. This same effect is also observed in the context of parental autonomy support (Girelli et al., 2018). Furthermore, parental involvement in educational aspirations is a strong predictor of academic self-efficacy in students (Fan & Williams, 2010). When parents encourage their children to make decisions—whether regarding sports choices, coursework, or managing academic and interpersonal issues—students tend to be more proactive in their academic pursuits, with a more pronounced effect in females (Pedersen, 2017). Academic self-efficacy is closely linked to emotional engagement in physical education. Students with high academic self-efficacy are more likely to exert effort in learning and actively seek opportunities and resources to improve (Gebauer et al., 2020). This includes participating in physical education classes and extracurricular sports training, such as seeking advice from teachers or professional coaches to correct technical errors (Shang et al., 2023). Conversely, students with low academic self-efficacy often lack confidence and display indifference in class (Bassi et al., 2007), which can lead to absenteeism or disengagement (Saeki & Quirk, 2015). Therefore, we hypothesize the following:

**H3a.**  *Academic self-efficacy mediates the relationship between teacher autonomy support and emotional engagement in physical education*.

**H3b.**  *Academic self-efficacy mediates the relationship between parental autonomy support and emotional engagement in physical education*.

### 2.4. The Chain Mediation Effect of Self-Acceptance and Academic Self-Efficacy

After analyzing the importance of autonomy support in emotional engagement in physical education, we hypothesize that self-acceptance and academic self-efficacy are interrelated and may contribute to positive engagement in physical education among university students. Research has shown that increased self-acceptance is associated with greater academic confidence, which in turn reduces academic burnout (Song, 2023). Another study indicated that parental warmth and reduced control can enhance children’s self-acceptance, thereby boosting their academic self-efficacy and reducing procrastination behaviors (Peng, 2022). Self-determination theory outlines the relationship between teacher autonomy support and student participation in learning activities (Sadoughi & Hejazi, 2021). In physical education, students who perceive greater autonomy support from their teachers tend to follow instructional norms more closely, actively engage in class, and express themselves more openly (Pan, 2014). This sense of competence fosters positive behavior and emotional involvement in class, leading to better academic performance (Olivier et al., 2019). Given these findings, it is plausible that self-acceptance and academic self-efficacy may mediate the relationship between autonomy support and emotional engagement in physical education. Thus, we propose the following hypotheses:

**H4a.**  *Self-acceptance and academic self-efficacy have a chain mediation effect between teacher autonomy support and emotional engagement in physical education*.

**H4b.**  *Self-acceptance and academic self-efficacy have a chain mediation effect between parental autonomy support and emotional engagement in physical education*.

**Figure 1:**
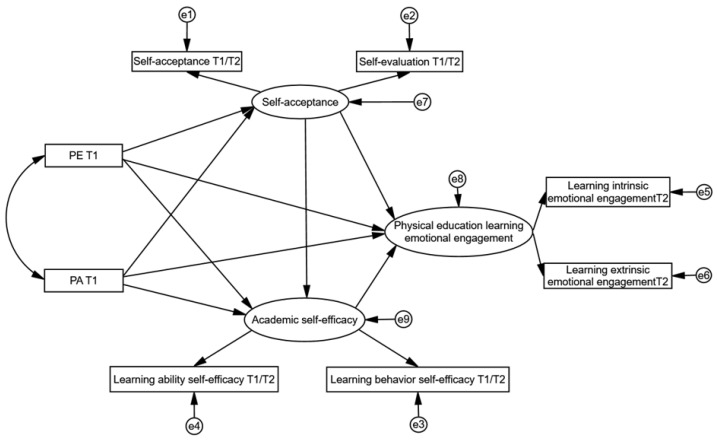
Theoretical hypothesis model.

Despite existing studies identifying a relationship between autonomy support and student engagement, research on the dynamic influence of external factors on students’ emotional engagement remains insufficient, especially in the context of physical education (Rodriguez Macias et al., 2021). Notably, current studies have not confirmed the connection between autonomy support and emotional engagement in physical education specifically. Additionally, the underlying mechanisms through which this relationship operates are still unclear. Understanding how these factors can beneficially affect emotional engagement in physical education is an important gap in research. It is also essential to recognize that one of the key attributes of engagement is its dynamic nature (Hiver et al., 2024). Emotional engagement can fluctuate subtly over the course of a semester, and much of the existing research may rely on cross-sectional surveys, which may overlook the continuity and stability of emotional engagement, thus affecting the accuracy of the results. To address this gap, our study employs a longitudinal design with university students to examine the chain mediation effect of self-acceptance and academic self-efficacy. The aim is to provide physical education practitioners with a deeper understanding of the significance of autonomy support in fostering effective student engagement. Figure 1 presents the hypothesized model for testing the above relationships.

## 3. Methodology

### 3.1. Measurement Subjects

This study employed cluster sampling to select university students from two universities in Ankang City, Shaanxi Province, China—Ankang University and Ankang Vocational and Technical College—as the subjects of the research. Ankang City is located in the remote southwestern region of Shaanxi Province, in northwest China. Due to differences in resource endowment and geographic conditions, the economic development level in this region lags behind, and its educational standards are relatively low. Choosing this region as the study site is of practical significance, as it provides insights into areas with less access to educational resources.

In most Chinese higher education institutions, students are typically required to go on internships during their senior year to accumulate practical experience. This requirement could interfere with long-term, fixed-point follow-up research. Therefore, this study focused on freshmen, sophomores, and juniors, conducting a 6-month longitudinal tracking survey. The first measurement (T1) was conducted in August 2024, with 942 valid participants. The second measurement (T2) was conducted in January 2025. Due to course adjustments, student absences, and illness, 224 participants were lost during the second measurement, resulting in 718 valid participants who completed both tests.

The average age of the participants was 19.98 years (standard deviation = 0.726). Additional demographic information on the participants can be found in Table 1. Analysis of the dropped-out and retained samples revealed no significant differences between the two groups in key variables, including teacher autonomy support, parental autonomy support, self-acceptance, academic self-efficacy, and emotional engagement in physical education (*p* > 0.05). The study followed strict procedures to ensure confidentiality, and approval was obtained from the Ankang University Review Committee. All participants signed informed consent forms before taking part in the study.

### 3.2. Research Tools

#### 3.2.1. Autonomous Support Questionnaires

The measurement tools for autonomous support in this study consist of two questionnaires: one assessing teacher support in physical education and the other evaluating parental autonomous support. The Learning Climate Questionnaire (LCQ), developed by Williams and Deci in 1996, measures perceived teacher autonomy support in specific learning contexts, typically applied to university students (E. Deci, 1996). This 15-item questionnaire uses a 7-point Likert scale, with scores ranging from 1 (strongly disagree) to 7 (strongly agree), where higher scores indicate greater perceived teacher support. Given the questionnaire’ s general application, it was revised to better align with physical education contexts (e.g., “My physical education teacher offers me multiple choices” and “My teacher encourages me to express my ideas”). In this study, the Cronbach’ s α values were 0.953 and 0.954 at two measurement points.

The parental autonomy support questionnaire was adapted from the scale by Tang et al. (Tang et al., 2013). Comprising 12 items (e.g., “When making decisions that affect me, my parents encourage me to express my opinions”), it uses a 5-point Likert scale (1 = strongly disagree, 5 = strongly agree), with higher scores reflecting greater parental autonomy support. In this study, the Cronbach’ s α values were 0.941 and 0.947 at two measurement points.

#### 3.2.2. Self-Acceptance Questionnaire

The Self-Acceptance Questionnaire (SAQ) was developed by Cong and Gao (Cong & Gao, 1999). The SAQ consists of two factors, self-acceptance and self-evaluation, with a total of 16 items (e.g., “I learn new things faster than others”). Each factor comprises 8 items, rated on a 4-point scale (1 = strongly disagree, 4 = strongly agree), with reverse-scored items adjusted to maintain consistency. Higher scores indicate greater self-acceptance. The Cronbach’ s α values at two time points were 0.935 and 0.932.

#### 3.2.3. Academic Self-Efficacy Questionnaire

The Academic Self-Efficacy Questionnaire was adapted from the scale by Pintrich and DeGroot (Pintrich & De Groot, 1990). The scale was revised by Liang Yusong for the Chinese context and further modified to suit physical education settings. It includes two dimensions: learning ability self-efficacy (11 items, e.g., “I believe I can achieve good results in physical education”) and learning behavior self-efficacy (11 items, e.g., “I independently practice physical skills outside class to assess my mastery”). A total of 22 items are rated on a 5-point Likert scale (1 = strongly disagree, 5 = strongly agree), with reverse scoring for items 14, 16, 17, and 20. Higher scores reflect greater academic self-efficacy. The Cronbach’ s α values at two time points were 0.962 and 0.962.

#### 3.2.4. Sports Learning Emotional Engagement Questionnaire

This questionnaire was developed by Guo Jidong (Guo, 2018), based on research by Appleton et al. (Appleton et al., 2006) and Fredricks and McColskey (Fredricks & McColskey, 2012). Through interviews and input surveys, it contains 24 entries, including intrinsic emotional investment in learning and extrinsic emotional investment in learning. The initial measurement KMO is 0.77, the Cronbach’s alpha coefficient was 0.89, and the structural model AIC values were all small and met the criteria. In order to conform to the environment and professional requirements of the physical education discipline, the expressions related to English learning in the questionnaire were revised to physical education classroom learning (e.g., “I think it is important to learn physical education knowledge and skills” and “I am confident in completing all physical education learning tasks”). As the questionnaire involved two related questions from the textbook that could not be adapted, they were deleted, and the questionnaire ended up containing a total of 22 entries. The Likert 5-point scale was used (from “1 = not at all” to “5 = completely”), and the higher the score, the higher the individual’s emotional commitment to physical education. The Cronbach’s alpha coefficients for the scale administered at the two time points in this study were 0.968 and 0.971.

### 3.3. Data Analysis

Data were processed and analyzed using SPSS 27.0 and Origin 2024 for descriptive statistics and correlation analysis. A mediating model was constructed using AMOS 24.0 to examine longitudinal relationships between variables, with a focus on the role of self-acceptance and academic self-efficacy.

## 4. Results and Analysis

### 4.1. Reliability and Validity Testing

To ensure the feasibility of the study, reliability and validity analyses were conducted using SPSS 27.0 and AMOS 24.0. The Cronbach’s α coefficients for the subscales ranged from 0.910 to 0.953, indicating high internal consistency. Convergent validity and composite reliability, calculated using Exps software tool, showed construct reliability values for all latent variables ranging from 0.909 to 0.956, exceeding the recommended threshold of 0.7, thus confirming the good internal consistency of the measurement model. Additionally, average variance extracted (AVE) values were computed, revealing that, except for academic behavior self-efficacy at Time 2 (AVE = 0.495), which remains within an acceptable range, all other dimensions had AVE values greater than 0.5, indicating ideal convergent validity. Overall, model fit indices suggest that the measurement models exhibit good fit with the data, confirming strong reliability and validity (Table 2).

### 4.2. Common Method Bias Test

This study first applied procedural controls (including randomization of questionnaire order, reverse scoring, and ensuring respondent anonymity) to reduce participants’ ability to guess the research purpose (Zhou & Long, 2004). In addition, Harman’s single-factor test was used to assess common method bias in the data. The results indicated that at both T1 and T2, nine factors had eigenvalues greater than 1. The variance explained by the first factor was 38.826% at T1 and 39.638% at T2, both below the critical threshold of 40%. Therefore, no significant common method bias was found in the data.

### 4.3. Longitudinal Measurement Invariance Test

This study tested the measurement invariance across two time points, including configural invariance, weak invariance (factor loadings invariance), and strong invariance (intercept invariance). Following the recommendations of Chen (Chen, 2007), measurement invariance was considered supported if the differences in fit indices were below the established thresholds (CFI change < 0.01, RMSEA change < 0.005). As shown in Table 3, while the measurement tools did not demonstrate intercept invariance, they did demonstrate factor loading invariance, indicating weak invariance.

### 4.4. Descriptive Statistics and Correlation Analysis

Descriptive statistics were used to analyze the mean values and standard deviations of each variable, as follows: PE Teacher Support (T1) = 74.28 ± 13.77, PE Teacher Support (T2) = 74.26 ± 14.20; Parental Autonomous Support (T1) = 47.67 ± 8.96, Parental Autonomous Support (T2) = 47.56 ± 8.90; Self-Acceptance (T1) = 17.61 ± 5.11, Self-Acceptance (T2) = 17.63 ± 5.01; Self-Assessment (T1) = 24.31 ± 5.34, Self-Assessment (T2) = 24.45 ± 5.13; Academic Self-Efficacy (T1) = 43.85 ± 8.28, Academic Self-Efficacy (T2) = 43.74 ± 8.36; Academic Behavior Self-Efficacy (T1) = 37.89 ± 8.32, Academic Behavior Self-Efficacy (T2) = 37.85 ± 8.17; Intrinsic Emotional Engagement in PE (T1) = 41.83 ± 8.64, Intrinsic Emotional Engagement in PE (T2) = 41.73 ± 9.89; Extrinsic Emotional Engagement in PE (T1) = 41.89 ± 8.28, Extrinsic Emotional Engagement in PE (T2) = 41.85 ± 8.61.

**Figure 2:**
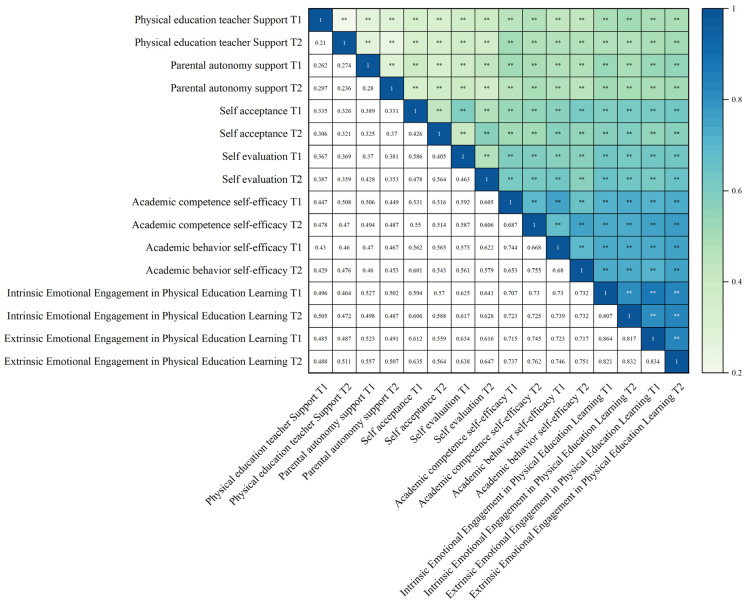
Correlation analysis of variables. ** *p* < 0.01.

Figure 2 presents the correlation analysis results. The findings indicate significant positive correlations at both time points: PE Teacher Support and Parental Autonomous Support were positively correlated with both Intrinsic and Extrinsic Emotional Engagement in PE, as well as with Self-Acceptance, Self-Assessment, Academic Behavior Self-Efficacy, and Academic Self-Efficacy. Self-Acceptance was positively correlated with Academic Behavior Self-Efficacy and Academic Self-Efficacy. Moreover, Self-Acceptance and Self-Assessment showed significant positive correlations with Academic Behavior Self-Efficacy, while Academic Self-Efficacy was positively related to both Intrinsic and Extrinsic Emotional Engagement in PE. These results provide preliminary support for the study hypotheses.

### 4.5. Longitudinal Mediation Analysis

**Figure 3:**
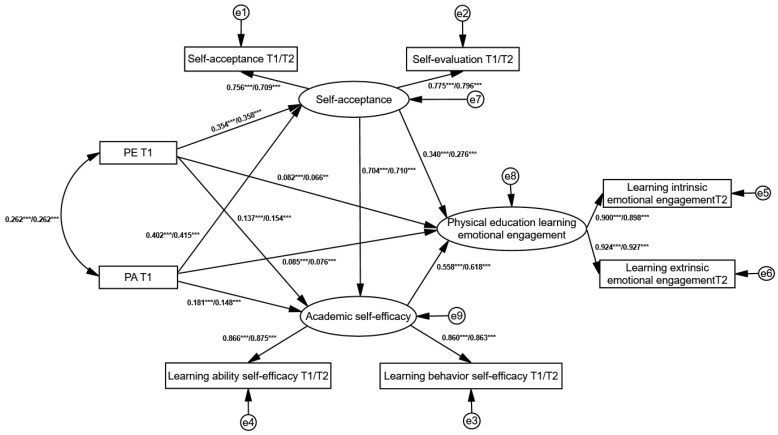
Longitudinal mediation model of self-acceptance and academic self-efficacy. (The coefficients shown represent the following: the left side represents the path coefficients when T1 Self-Acceptance and T1 Academic Self-Efficacy serve as mediators, while the right side represents the path coefficients when T2 Self-Acceptance and T2 Academic Self-Efficacy serve as mediators. For clarity and brevity, the path coefficients for covariates are not labeled on the diagram; the same applies to subsequent figures. *** *p* < 0.001, ** *p* < 0.01).

First, we tested the immediate mediation effects at both time points. Building on this, we further examined the longitudinal mediation effects of Self-Acceptance and Academic Self-Efficacy across time. The analysis involved T1 PE Teacher Support and T1 Parental Autonomous Support as predictors, with T1 and T2 Self-Acceptance and Academic Self-Efficacy as mediators, and T2 Emotional Engagement in PE as the outcome variable, constructing a longitudinal mediation model. The results indicated good model fit for both models: When T1 Self-Acceptance and Academic Self-Efficacy were mediators: χ^2^/df = 2.041, NFI = 0.993, RFI = 0.984, IFI = 0.997, TLI = 0.992, CFI = 0.997, RMSEA = 0.038. When T2 Self-Acceptance and Academic Self-Efficacy were mediators: χ^2^/df = 2.857, NFI = 0.991, RFI = 0.978, IFI = 0.994, TLI = 0.986, CFI = 0.994, RMSEA = 0.051. The model results (see Figure 3) revealed the following when T1 Self-Acceptance and Academic Self-Efficacy were mediators:

T1 PE Teacher Support and T1 Parental Autonomous Support both significantly positively predicted T1 Self-Acceptance (*β* = 0.354, *p* < 0.001; *β* = 0.402, *p* < 0.001). T1 PE Teacher Support and T1 Parental Autonomous Support both significantly positively predicted T1 Academic Self-Efficacy (*β* = 0.137, *p* < 0.001; *β* = 0.181, *p* < 0.001). T1 Self-Acceptance significantly positively predicted T1 Academic Self-Efficacy (*β* = 0.704, *p* < 0.001). T1 PE Teacher Support and T1 Parental Autonomous Support both significantly positively predicted T2 Emotional Engagement in PE (*β* = 0.082, *p* < 0.001; *β* = 0.076, *p* < 0.001). T1 Self-Acceptance and T1 Academic Self-Efficacy both significantly positively predicted T2 Emotional Engagement in PE (*β* = 0.340, *p* < 0.001; *β* = 0.558, *p* < 0.001). When T2 Self-Acceptance and T1 Academic Self-Efficacy served as mediators, the results were consistent with those described above. Specific path coefficients are presented in Table 4.

Based on the good model fit and significant direct paths in the longitudinal mediation model, we further validated the chain mediation effect of Self-Acceptance and Academic Self-Efficacy using the bootstrap method with 5000 resamples. The standardized estimates for each indirect path and the 95% confidence intervals for the mediation effects are presented in Table 5.

The results showed the following: The mediation effects of T1 Self-Acceptance and T1 Academic Self-Efficacy between T1 PE Teacher Support and T2 Emotional Engagement in PE were 0.070 (95% CI [0.04, 0.10]) and 0.044 (95% CI [0.02, 0.07]), respectively, both not including zero, accounting for 28.92% and 18.18% of the total effect. The chain mediation effect of T1 Self-Acceptance and T1 Academic Self-Efficacy was significant, with an effect size of 0.080 (95% CI [0.05, 0.11]), accounting for 33.05% of the total effect. The mediation effects of T1 Self-Acceptance and T1 Academic Self-Efficacy between T1 Parental Autonomous Support and T2 Emotional Engagement in PE were 0.130 (95% CI [0.07, 0.19]) and 0.096 (95% CI [0.05, 0.14]), respectively, both not including zero, accounting for 26.97% and 19.91% of the total effect. The chain mediation effect of T1 Self-Acceptance and T1 Academic Self-Efficacy was significant, with an effect size of 0.150 (95% CI [0.10, 0.20]), accounting for 31.12% of the total effect. When T2 Self-Acceptance and T2 Academic Self-Efficacy served as mediators, the results were consistent with those described above. Specific path coefficients are shown in Table 5. The findings suggest the following: Self-Acceptance and Academic Self-Efficacy independently mediate the effects of PE Teacher Support and Parental Autonomous Support on college students’ Emotional Engagement in PE. Self-Acceptance and Academic Self-Efficacy both independently and jointly mediate the effects of PE Teacher Support and Parental Autonomous Support on Emotional Engagement in PE, with no significant difference in the magnitude of the independent mediation effects.

## 5. Discussion

The primary aim of this study was to test the predictive model of autonomy support on college students’ emotional engagement in physical education. The model employed a longitudinal design, using data collected at two time points: the beginning and the end of the semester. While previous research has explored the relationship between supportive environments and learning engagement, the longitudinal changes in students’ emotional engagement in physical education, particularly in the context of a specialized environment, have not been fully investigated. This study’s structural model verified and analyzed the longitudinal and chain mediation effects of Self-Acceptance and Academic Self-Efficacy. This design offers valuable insights for educators on how to implement autonomy-supportive strategies and predict students’ emotional engagement in physical education following teacher and parental support, validated through our follow-up survey.

The first hypothesis confirmed that both PE Teacher Support and Parental Autonomous Support significantly influenced college students’ emotional engagement in physical education. This finding aligns with Self-Determination Theory (SDT) (Kaplan, 2018). The longitudinal data study found that college students’ affective engagement in physical education learning in different autonomy support environments showed stable baseline levels over time, implying that either physical education teacher support or parental autonomy support significantly promoted college students’ affective engagement in physical education learning during the semester teaching phase. College is a critical period for growth and development, during which students face various academic challenges and emotional experiences (Qiu & Ye, 2023). Perceptions of autonomy support from PE teachers contribute to a more active classroom atmosphere, enhancing students’ interest and enjoyment in physical education (Ommundsen & Kval⊘, 2007). In traditional PE education, which is often teacher-centered and performance-oriented, students’ learning is primarily evaluated based on technical proficiency or performance outcomes, without much emphasis on decision-making or problem-solving (C. Liu, 2023).

Ecological dynamics and systems theory propose a framework for guiding strategies (Woods et al., 2020), where educators encourage learners to explore and discover solutions through prompts, questions, and task constraints rather than step-by-step guidance (Großmann & Wilde, 2019). In an autonomy-supportive teaching environment, students feel less pressured and are more likely to engage voluntarily in their learning, completing tasks in their own way, learning self-reflection, and becoming less reliant on traditional teacher instruction. This approach fosters deeper engagement and self-directed learning. However, it should not be overlooked that pre-service training is an important process for developing support for teacher autonomy. Continuing development training allows teachers to understand how to stimulate the classroom climate to better promote student professional development (Girard & De Guise, 2024). Therefore, the study also highlights the need for physical education teachers to consolidate classroom autonomy support from training to give more dynamism to the classroom. Additionally, our findings emphasized the significant role of parental autonomy support in students’ emotional engagement in physical education. In the context of China’s exam-oriented education system, obtaining academic credits is crucial for graduation, often driving students’ learning behaviors. This credit-driven model can limit opportunities for self-exploration and personal growth during physical education classes. In contrast, in developed countries such as in Europe, universities and sports institutions often collaborate with parents through educational models like EMPATIA, where parents provide emotional, logistical, and financial support to balance their children’s academic and athletic pursuits (Gjaka et al., 2021). This type of support enables students to engage more fully in both training and learning. For example, autonomy support provided by parents who demonstrate a small amount of pressure and expectations can lead to more spontaneous and natural behavior on the court (Gillet et al., 2012). Therefore, based on international best practices, we recommend that Chinese universities consider strengthening parental support strategies to foster students’ emotional engagement in physical education. Such strategies could enhance students’ holistic development, providing greater flexibility for their future career planning and well-being.

Through mediation analysis, we found that self-acceptance and academic self-efficacy play a longitudinal mediating role between autonomy support and emotional engagement in physical education. The more autonomy support students perceive from physical education teachers and parents, the higher their self-acceptance, thereby strengthening their emotional engagement in physical learning. Self-acceptance not only directly predicts individual developmental outcomes but also serves as a mediator between external factors and these outcomes. Studies show that autonomy-supportive practices at home and school can help students cultivate self-acceptance (X. Zhang et al., 2022). Autonomy-supportive teachers, who consider students’ opinions, interests, and concerns, foster an environment where students feel that their choices are independent, enabling selective learning based on personal circumstances and the specifics of different sports (Yu et al., 2016). This approach creates an atmosphere of self-directed exploratory learning in the classroom.

Higgins’ self-discrepancy theory divides the self into actual, ideal, and ought selves, positing that dissatisfaction with the actual self may lead to neglect of positive self-aspects (Higgins, 1987). Self-acceptance helps individuals acknowledge and affirm their worth, face their realities, and reduce frustration and anxiety, thereby promoting emotional well-being (Toyota, 2011). This also applies in classroom learning, where higher levels of self-acceptance correlate with positive emotions such as hope, joy, and interest (Jimenez et al., 2010). Thus, when students encounter setbacks in physical education, they are more likely to acknowledge and embrace these negative stimuli, adopting positive coping strategies. Conversely, low self-acceptance leads to lack of motivation and feelings of helplessness, often resulting in anxiety and stress (Kim et al., 2018). Regarding parental autonomy support, respectful and supportive parenting enhances self-acceptance, offering students the chance to think critically and resolve problems independently, accepting their imperfections (Kanakia & Bhansali, 2024). Developmental-contextual theory posits that individual development is shaped by the interaction between the person and the environment (Lerner et al., 2006). Specifically, after receiving parental support, students gain confidence, overcome challenges, and develop a more immersive learning experience in physical education. However, due to cultural differences between Western and Chinese societies, where parental relationships emphasize “filial piety,” Chinese parenting tends to be more controlling, protective, and indulgent. This often leads students to seek parental validation during physical skills acquisition, potentially causing negative psychological outcomes such as depression and anxiety. In such contexts, parents need to encourage greater autonomy, enabling students to develop a more positive self-concept and self-evaluation, thus fostering individual growth in physical education, training, and competition. In Western countries, there is a greater emphasis on individualism, an independent self-concept, privacy, personal space, and self-expression (Markus & Kitayama, 1991). Thus culture, education model, personal experience, and learning environment do have a certain impact on the development of students’ affective learning skills. In this regard, the cultural differences between Western and Chinese education need to be taken into account in order to tailor teaching and learning programs.

Additionally, our study confirmed the mediating role of academic self-efficacy. Motivation theory suggests that learning behavior results from the interaction of external factors, individual cognition, and motivation (Schweder, 2019). Students who attribute learning outcomes to controllable internal factors exhibit better self-regulation and monitoring of their learning (Shilenkova, 2020). Research shows that students who perceive more autonomy support from their teachers not only complete assigned tasks but also willingly take on more challenging tasks, thus acquiring more knowledge and skills (Martin & Dowson, 2009). This is crucial in physical education, where, despite initial enthusiasm, students’ engagement tends to decrease as their effort wanes over the term (Oga-Baldwin et al., 2021). Autonomy-supportive teachers, who enrich learning materials, listen to students’ opinions, and create dynamic, supportive environments, can counteract this decline and enhance enthusiasm and learning outcomes. In China, physical education courses are divided into general and specialized tracks, with curricula designed for different student populations. While students in physical education majors often show superior athletic skills, non-major students tend to be less skilled, requiring greater effort toward the end of the term. Most teachers provide review outlines, which may lead to student disengagement or negative feelings toward physical education. Therefore, teachers should consider implementing autonomy-supportive strategies. Ecological systems theory emphasizes the role of the family environment as a micro-support system for individual development. A supportive learning environment, including strong relationships among family, teachers, and peers, fosters intrinsic motivation and proactive learning (Kaplan, 2018). For instance, when parents convey the value of physical health, they help students recognize the importance of physical education for future academic and career success, thereby enhancing students’ self-directed learning and perseverance.

Finally, the results of the study showed that self-acceptance and academic self-efficacy played an important role in the chain of transmission between them, which accounted for a significant proportion of the total effect and also indicated a high correlation between the two. It has also been found that the higher the social support and self-acceptance, the higher the student’s self-confidence on campus (Pastimo & Muslikah, 2022), which may show a more positive emotional experience in classroom learning. Our study found that either physical education faculty support or parental autonomy support promoted college students’ affective engagement in physical education learning through enhanced self-acceptance and increased academic self-efficacy. Importantly, this effect was significantly associated with a longitudinal design, which has not been found in previous studies. It can be explained by the fact that a supportive teacher or home environment allows students to feel a greater sense of freedom and choice, which leads to a greater sense of self-acceptance (Wu et al., 2024). Whereas high self-acceptance helps individuals to build trust in themselves, accept their imperfections, and reduce excessive worry about failing to learn physical skills, this may increase confidence to commit to physical education, which in turn may result in higher levels of positive affect and engagement.

This study has some contributory value. The use of longitudinal data to avoid the limitations of static cross-sections better reveals the causal mechanism between autonomous hosting and affective engagement in physical education learning, providing insights for educational practitioners. In addition, the self-determination theory was extended and refined, and self-acceptance, as a core embodiment of autonomy needs, formed a synergistic mechanism with academic self-efficacy, which together explained the sustainability of students’ affective engagement in physical education learning under the SDT framework. This study has limitations that suggest directions for future research. First, we assessed the relationship between autonomy support and emotional engagement at two time points using self-reported data. While statistically significant, we could not observe the impact of other factors, such as academic overload or injuries. In future studies, we will incorporate more observational factors, screen the sample more carefully, and extend the time point of the test to make the study more scientifically valid. Furthermore, we could not control for the teaching content, methods, or approaches, as these are dictated by the academic calendar. Therefore, teaching content may serve as a confounding variable, and future research should compare different supportive environments. For example, during game sessions or competitions in the physical education classroom, the trajectory of students’ affective engagement can be observed to provide insights for the efficient development of teaching programmes. Finally, sample attrition during the second measurement may have influenced the results, as dropouts and other factors like illness or transfers may limit the findings. Future studies should consider surveying students from diverse regions to explore regional differences in emotional engagement in physical education.

## 6. Conclusions

This study, based on longitudinal survey data, examined the impact of different types of autonomy support—specifically, support from physical education teachers and parents—on university students’ emotional engagement in physical education. We also tested the longitudinal independent and chain mediation effects of self-acceptance and academic self-efficacy. Our findings indicate that both teacher and parental autonomy support positively enhance students’ emotional engagement in physical education, offering practical strategies to improve the quality of physical education programs. Furthermore, self-acceptance and academic self-efficacy play crucial roles as mediators in this relationship, with their effects remaining significant over time. For more precise inferences regarding the relationship between autonomy support and emotional engagement in physical education, future longitudinal studies should consider extending the measurement intervals. Additionally, examining the impact of autonomy-supportive strategies across different academic years and assessing students’ athletic performance under these influences will provide a more nuanced understanding of the evolving patterns in students’ emotional engagement in physical education.

## Figures and Tables

**Table 1:** Basic information of the participants (N = 718).

Characteristic	Category	Frequency	Percentage
Gender	Female	349	48.6%
	Male	369	51.4%
Grade	Freshman	230	32%
	Sophomore	232	32.3%
	Junior	256	35.7%
Household Registration	Rural	248	34.5%
	Urban	470	65.5%
Only Child	No	261	36.4%
	Yes	457	63.6%
Physical Education Major	No	515	71.7%
	Yes	203	28.3%

**Table 2:** Convergent validity and composite reliability tests for each variable.

Variable	Standardized Loadings	*a*	CR	AVE
Autonomous Support				
PE Teacher Support (T1)		0.953	0.956	0.611
PE1	0.815			
PE2	0.806			
PE3	0.802			
PE4	0.795			
PE5	0.793			
PE6	0.789			
PE7	0.789			
PE8	0.788			
PE9	0.784			
PE10	0.781			
PE11	0.779			
PE12	0.774			
PE13	0.767			
PE14	0.672			
Parental Autonomous Support (T1)		0.941	0.946	0.597
PA1	0.785			
PA2	0.783			
PA3	0.781			
PA4	0.778			
PA5	0.777			
PA6	0.777			
PA7	0.773			
PA8	0.771			
PA9	0.766			
PA10	0.764			
PA11	0.763			
PA12	0.758			
Self-Assessment (T1/T2)		0.926 (0.923)	0.922 (0.921)	0.597 (0.594)
SE1	0.795(0.798)			
SE2	0.787(0.789)			
SE3	0.779(0.777)			
SE4	0.775(0.776)			
SE5	0.767(0.772)			
SE6	0.766(0.756)			
SE7	0.758(0.750)			
SE8	0.756(0.749)			
Self-Acceptance (T1/T2)		0.911 (0.910)	0.909 (0.910)	0.558 (0.560)
SA1	0.797(0.784)			
SA2	0.793(0.781)			
SA3	0.781(0.763)			
SA4	0.762(0.751)			
SA5	0.758(0.747)			
SA6	0.742(0.743)			
SA7	0.683(0.740)			
SA8	0.651(0.675)			
Academic Ability Self-Efficacy (T1/T2)		0.947 (0.948)	0.930 (0.931)	0.548 (0.551)
AC1	0.768(0.762)			
AC2	0.766(0.761)			
AC3	0.762(0.754)			
AC4	0.759(0.753)			
AC5	0.742(0.753)			
AC6	0.740(0.750)			
AC7	0.738(0.734)			
AC8	0.738(0.731)			
AC9	0.713(0.728)			
AC10	0.712(0.724)			
AC11	0.708(0.714)			
Academic Behavior Self-Efficacy (T1/T2)		0.941 (0.934)	0.922 (0.914)	0.518 (0.495)
AB1	0.761(0.730)			
AB2	0.754(0.719)			
AB3	0.751(0.716)			
AB4	0.733(0.711)			
AB5	0.723(0.710)			
AB6	0.720(0.703)			
AB7	0.703(0.701)			
AB8	0.700(0.700)			
AB9	0.694(0.694)			
AB10	0.694(0.679)			
AB11	0.685(0.670)			
Intrinsic Emotional Engagement in PE (T2)		0.944	0.925	0.554
IE1	0.766			
IE2	0.759			
IE3	0.757			
IE4	0.756			
IE5	0.751			
IE6	0.750			
IE7	0.748			
IE8	0.741			
IE9	0.712			
IE10	0.702			
Extrinsic Emotional Engagement in PE (T2)		0.946	0.925	0.508
EL1	0.782			
EL2	0.743			
EL3	0.734			
EL4	0.727			
EL5	0.716			
EL6	0.714			
EL7	0.709			
EL8	0.707			
EL9	0.702			
EL10	0.697			
EL11	0.664			
EL12	0.652			

**Table 3:** Longitudinal measurement invariance test for each scale.

Variable	χ^2^	df	CFI	TLI	RMSEA	ΔCFI	ΔRMSEA
PE Teacher Support							
Configural Invariance	378.357	335	0.995	0.995	0.013	-	-
Weak Invariance	426.208	348	0.991	0.991	0.018	−0.004	0.005
Strong Invariance	2388.130	376	0.778	0.777	0.086	−0.217	0.073
Parental Autonomy Support							
Configural Invariance	225.807	239	1.000	1.002	0.000	-	-
Weak Invariance	246.182	250	1.000	1.001	0.000	0.000	0.000
Strong Invariance	814.793	274	0.934	0.933	0.052	−0.066	0.052
Self-acceptance							
Configural Invariance	562.923	442	0.989	0.988	0.020	-	-
Weak Invariance	588.334	456	0.988	0.987	0.020	−0.001	0.000
Strong Invariance	3874.799	488	0.703	0.698	0.098	−0.029	0.078
Academic Self-efficacy							
Configural Invariance	1258.557	874	0.978	0.976	0.025	-	-
Weak Invariance	1296.023	894	0.977	0.975	0.025	−0.001	0.000
Strong Invariance	3637.430	937	0.843	0.842	0.063	−0.135	0.038
PE Learning Emotional Engagement							
Configural Invariance	2524.444	965	0.921	0.915	0.047	-	-
Weak Invariance	2589.883	987	0.919	0.915	0.048	−0.002	0.001
Strong Invariance	4883.011	1033	0.805	0.804	0.072	−0.116	0.025

**Table 4:** Direct path effect tests in the longitudinal mediation model.

Time Point	Standardized Loading	Standard Error	CR	Hypothesis Test Result
T1 Self-acceptance and Academic Self-efficacy				
PE Teacher Support T1→Self-acceptance T1	0.354	0.011	8.915	Supported
Parental Autonomy Support T1→Self-acceptance T1	0.402	0.018	10.042	Supported
PE Teacher Support T1→Academic Self-efficacy T1	0.137	0.018	3.996	Supported
Parental Autonomy Support T1→Academic Self-efficacy T1	0.181	0.031	12.757	Supported
Self-acceptance T1→Academic Self-efficacy T1	0.704	0.103	12.757	Supported
PE Teacher Support T1→PE Learning Emotional Engagement T2	0.082	0.014	3.431	Supported
Parental Autonomy Support T1→PE Learning Emotional Engagement T2	0.076	0.024	3.017	Supported
Self-acceptance T1→PE Learning Emotional Engagement T2	0.340	0.146	4.807	Supported
T2 Self-acceptance and Academic Self-efficacy				
PE Teacher Support T1→Self-acceptance T2	0.358	0.010	8.862	Supported
Parental Autonomy Support T1→Self-acceptance T2	0.415	0.017	10.114	Supported
PE Teacher Support T1→Academic Self-efficacy T2	0.154	0.018	4.357	Supported
Parental Autonomy Support T1→Academic Self-efficacy T2	0.148	0.031	4.010	Supported
Self-acceptance T2→Academic Self-efficacy T2	0.710	0.117	12.071	Supported
PE Teacher Support T1→PE Learning Emotional Engagement T2	0.066	0.014	2.738	Supported
Parental Autonomy Support T1→PE Learning Emotional Engagement T2	0.085	0.024	3.399	Supported
Self-acceptance T2→PE Learning Emotional Engagement T2	0.276	0.158	3.926	Supported
Academic Self-efficacy T2→PE Learning Emotional Engagement T2	0.618	0.082	8.513	Supported

**Table 5:** Mediation effects of self-acceptance and academic self-efficacy and 95% confidence intervals.

Path	Effect Value	Standard Error	Lower Bound	Upper Bound	Effect Size
Mediation Model T1					
Total Effect	0.242	0.018	0.206	0.277	100%
Direct Effect	0.047	0.014	0.019	0.075	19.42%
PE Teacher Support T1→Self-acceptance T1→PE Learning Emotional Engagement T2	0.070	0.017	0.040	0.106	28.92%
PE Teacher Support T1→Academic Self-efficacy T1→PE Learning Emotional Engagement T2	0.044	0.014	0.020	0.073	18.18%
PE Teacher Support T1→Self-acceptance T1→Academic Self-efficacy T1→PE Learning Emotional Engagement T2	0.080	0.014	0.057	0.113	33.05%
Total Effect	0.448	0.060	0.371	0.608	100%
Direct Effect	0.073	0.025	0.022	0.122	15.14%
Parental Autonomy Support T1→Self-acceptance T1→PE Learning Emotional Engagement T2	0.130	0.031	0.076	0.198	26.97%
Parental Autonomy Support T1→Academic Self-efficacy T1→PE Learning Emotional Engagement T2	0.096	0.024	0.053	0.148	19.91%
Parental Autonomy Support T1→Self-acceptance T1→Academic Self-efficacy T1→PE Learning Emotional Engagement T2	0.150	0.025	0.107	0.207	31.12%
Mediation Model T2					
Total Effect	0.242	0.018	0.206	0.277	100%
Direct Effect	0.039	0.014	0.011	0.067	16.11%
PE Teacher Support T1→Self-acceptance T2→PE Learning Emotional Engagement T2	0.057	0.018	0.028	0.098	23.55%
PE Teacher Support T1→Academic Self-efficacy T2→PE Learning Emotional Engagement T2	0.055	0.016	0.026	0.089	22.72%
PE Teacher Support T1→Self-acceptance T2→Academic Self-efficacy T2→PE Learning Emotional Engagement T2	0.091	0.016	0.065	0.127	37.60%
Total Effect	0.450	0.029	0.395	0.508	100%
Direct Effect	0.081	0.024	0.031	0.128	18%
Parental Autonomy Support T1→Self-acceptance T2→PE Learning Emotional Engagement T2	0.109	0.032	0.053	0.179	24.22%
Parental Autonomy Support T1→Academic Self-efficacy T2→PE Learning Emotional Engagement T2	0.087	0.027	0.038	0.146	19.33%
Parental Autonomy Support T1→Self-acceptance T2→Academic Self-efficacy T2→PE Learning Emotional Engagement T2	0.173	0.027	0.128	0.236	38.44%

## Data Availability

Data are available from the corresponding author upon reasonable request.
